# Serum Levels of Thyroid Hormones in Relation to Breast Cancer Prognosis: A Multicentre Cohort Study

**DOI:** 10.1002/cam4.72264

**Published:** 2026-09-09

**Authors:** Annie Brange, Ylva Heyman, Kamil Demircan, Johan Vallon‐Christersson, Martin Malmberg, Lao H. Saal, Lisa Rydén, Åke Borg, Lutz Schomburg, Jonas Manjer

**Affiliations:** ^1^ Department of Clinical Sciences Malmö Lund University Malmö Sweden; ^2^ Department of Ear‐, Nose‐ and Throat Skåne University Hospital Malmö Sweden; ^3^ Division of Oncology, Department of Clinical Sciences Lund Lund University, Medicon Village Lund SE Sweden; ^4^ Institute for Experimental Endocrinology Charité‐Universitätsmedizin Berlin, Corporate Member of Freie Universität Berlin, Humboldt‐Universität Zu Berlin, and Berlin Institute of Health Berlin Germany; ^5^ Department of Haematology, Oncology and Radiation Physics Skåne University Hospital Lund Sweden; ^6^ Division of Surgery, Department of Clinical Sciences Lund University Lund Sweden; ^7^ Department of Surgery Skåne University Hospital Malmö Sweden

**Keywords:** breast cancer, prognosis, thyroid hormones

## Abstract

Thyroid function has been suggested to be associated with breast cancer risk, but not much is known about thyroid hormones as a potential prognostic factor for breast cancer. This study investigated the relation between thyroid hormones in breast cancer patients and mortality. A total of 2000 breast cancer patients from the Swedish SCAN‐B cohort were followed for 9 years. Blood samples were collected at the time of diagnosis, before surgery, and analysed for free and total T3 and T4. All‐cause mortality and recurrent disease were compared between quartiles (Q) using Cox proportional hazard analysis adjusted for prognostic factors, yielding hazard ratios (HR) with 95% confidence intervals (CIs). During follow‐up, 310 deaths and 167 recurrences occurred. Free T3 was associated with a lower mortality; the combined group Q2 to Q4 had an adjusted HR of 0.73 (0.58–0.93) as compared to Q1. Moreover, a high ratio of free T3/T4 was associated with a low mortality in Q2, Q3 and Q4, respectively. High levels of free and total T4 were associated with a high risk of all‐cause mortality; the crude HR for free T4 Q4 vs. Q1 was 1.53 (1.14–2.07) and the corresponding HR for total T4 was 1.63 (1.20–2.22). These results did not remain in the adjusted analysis. No clear associations were found regarding recurrent disease. We conclude that high levels of free T3, and a high free T3/T4 ratio were associated with a lower risk of all‐cause mortality and may be a marker of a favourable prognosis.

## Graphical abstract

High free T3 levels at diagnosis are associated with good survival following breast cancer.

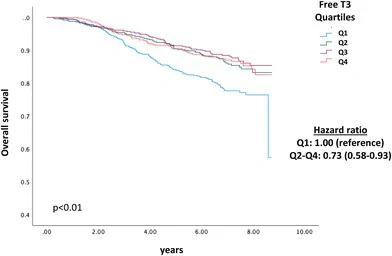

**Abbreviations**
CI: confidence interval
CIS: cancer in situ
CV: coefficients of variation
DIO1: iodothyronine deiodinase 1
E2: oestradiol
ER: oestrogen receptor
HR: hazard ratios
IHC: immunohistochemistry
ISH: in situ hybridization
NHG: Nottingham histological grade
NKBC: The Swedish Quality Register for Breast Cancer
PgR: progesterone receptor
Q: quartiles
RR: relative risk
SCAN‐B: Sweden Cancerome Analysis Network—Breast
SMRT: silencing mediator of retinoid and thyroid receptor
T3: triiodothyronine
T4: thyroxine
THR: thyroid hormone receptor

## 1 Introduction

Thyroid function has been suggested to play a role in breast cancer prognosis, but the evidence remains inconclusive [1, 2, 3, 4, 5]. Previous studies have mostly focused on measuring thyroid levels from patients with thyroid dysfunction or using information on hormonal levels obtained up to several years prior to the diagnosis of breast cancer.

Prognostic factors for breast cancer are used in clinical practise to predict survival, but also the risk of recurrent disease. Tumour stage, grade and proliferation (e.g., according to Ki67) are important prognostic factors and can be classified according to the TNM system [6]. Treatment predictive factors include oestrogen receptor (ER) and progesterone receptor (PgR) expression, along with amplification of the HER2 gene and expression of the proliferation marker Ki67 [7, 8].

The thyroid gland affects the breast through production of hormones which induce cell proliferation and modulate the effect of oestrogens on breast cell differentiation [9]. These factors contribute to normal tissue growth, but have also shown to be involved in breast cancer development [10]. Several proteins involved in the regulation of thyroid hormone activity have been seen on cancer cells. The thyroid hormone receptor (THR) has shown to be expressed in cancer cells, along with oestrogen receptors (ER) [11, 12]. In pre‐clinical studies, it has also been suggested that triiodothyronine (T3) can enhance and mimic the effects of oestradiol (E2) on breast cancer cell proliferation [2]. The DIO1 (Iodothyronine deiodinase 1) is another example of a protein expressed in cancer cells. DIO1 is responsible for the conversion of thyroxine (T4) to T3, i.e., the activation of the prohormone T4 to the active hormone tri‐iodothzronine [13]. Free and total thyroid hormone fractions are in equilibrium, and a change in the free T3/free T4 ratio will result in a change in the total T3/total T4 ratio under normal conditions, when the binding proteins are not severely affected. Demircan et al. found, indeed, that a high DIO1 mRNA expression in women with high serum selenium levels were associated with lower mortality in breast cancer [14].

A previous study by Brandt et al. has shown that high pre‐diagnostic levels of free T4 are associated with a higher breast cancer risk, although tumours may exhibit less aggressive characteristics [1, 15]. It is intriguing that the same factor may be a risk factor but also a favourable prognostic factor; this has also been observed for other breast cancer risk factors, and potential explanations include selection of tumour subgroups or multiple biological mechanisms. Another study by Tosovic et al. found a positive correlation between total T3 and breast cancer mortality, as well as unfavourable prognostic tumour characteristics [5, 16]. Studies investigating individuals with hypo‐ or hyperthyroidism have suggested an increased risk of breast cancer among those with hyperthyroidism and a decreased risk of breast cancer among those with hypothyroidism compared to the general population [17]. Furthermore, in an experimental study, Tamoxifen (commonly used in the treatment of breast cancer) was converted from a partial antagonist to an agonist of the ER in breast cancer cells, in the presence of exogenous thyroid hormones [18]. This contributes to disease progression, rather than suppression, and highlights the importance of further investigating the relationship between thyroid hormones and breast cancer, as this potential mechanism could lead to an effect where thyroid hormone levels would act as a treatment predictive factor.

Considering these facts about the involvement of the thyroid gland in breast cancer, we hypothesised that levels of thyroid hormones could predict outcome in breast cancer and function as a prognostic factor.

This study aimed to examine the levels of free and total thyroid hormones, T3 and T4, at the time of breast cancer diagnosis, and their associations with all‐cause mortality and risk of recurrent disease within a large multicentre cohort study. To our knowledge, this is the largest study of thyroid function on breast cancer mortality.

## 2 Methods

### 2.1 The SCAN‐B Cohort

In 2010 the Sweden Cancerome Analysis Network—Breast (SCAN‐B) was set up in the southern regions of Sweden. Participating hospitals included Lund, Malmö, Helsingborg, Halmstad, Karlskrona, Varberg and Ljungby. The aim was to analyse the genome of breast cancer in order to find more individualised treatment for each patient and thereby reducing mortality [19]. Patients have been enrolled since 30 August 2010. Enrolment was offered to patients during the clinical appointment when the patient was informed of the diagnosis and when treatment was planned.

Eligibility criteria for participation was a preoperative diagnosis of primary invasive breast cancer, preoperative suspicion of breast cancer, or neoadjuvant therapy following a diagnosis of breast cancer. Participating patients received the same standard of treatment as non‐participating patients. Patients had to be treated at one of the participating hospitals. Exclusion criteria involved generalised disease at diagnosis (i.e., distant metastases/stage IV disease), unknown treatment status relating to breast cancer, prior contralateral disease, or no primary surgery planned [19, 20]. Most patients were early cases, i.e., without lymph node metastases (64.2%).

### 2.2 Study Population

**FIGURE 1:**
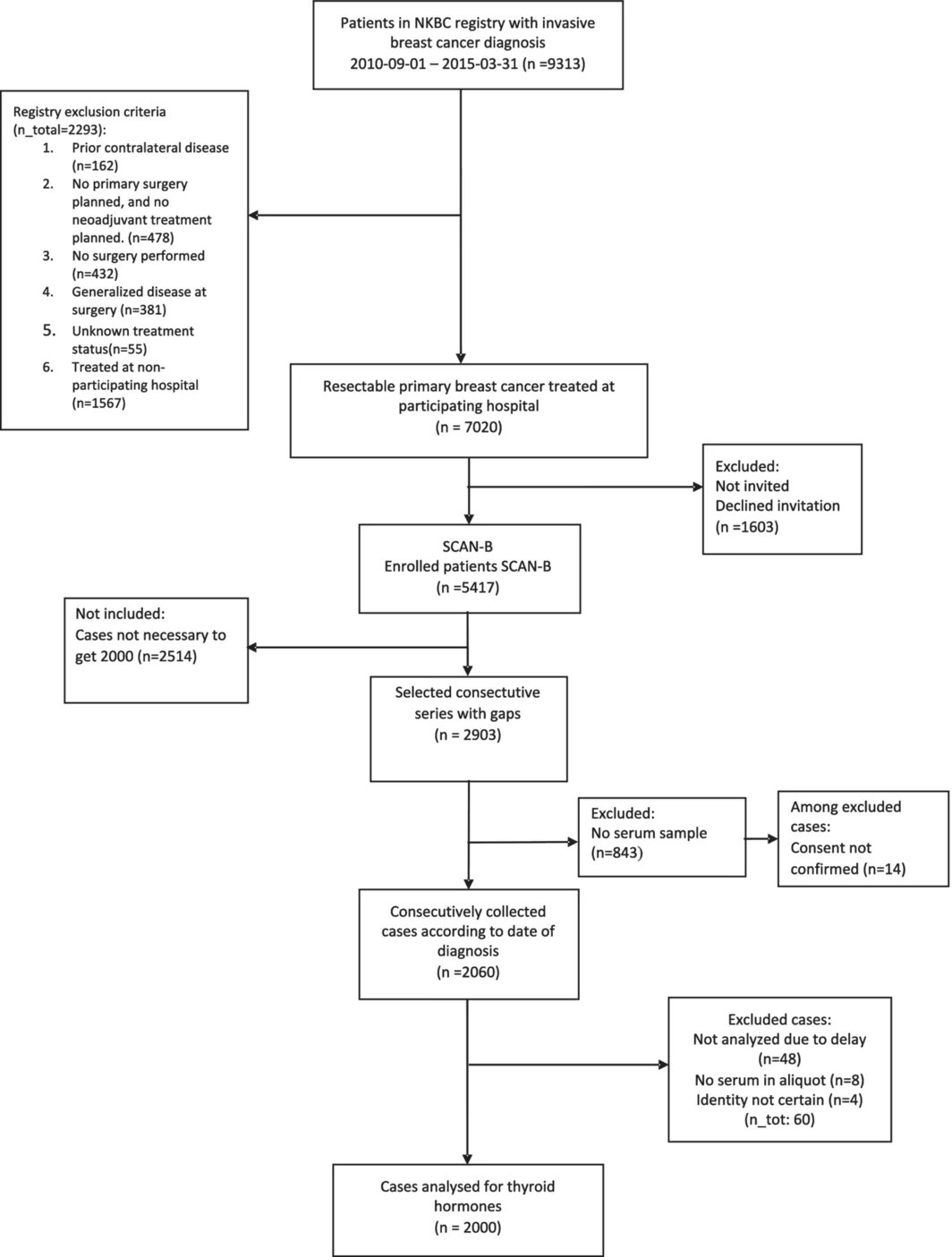
Flowchart of patient selection.

During the period September 2010 through March 2015 there were 9313 cases with invasive breast cancer in the Swedish Quality Register for Breast Cancer (NKBC) in the catchment area. Out of these, 5417 patients were included in the SCAN‐B study. For the purpose of the current study, all patients were given a number from 1 to 5417 and sorted in accordance with the date of diagnosis, from 2010 until 2015. The present study planned to include 2000 cases. Statistical power was calculated in the original project based on breast cancer specific mortality. Low serum levels were defined as the first quartile and used alpha: 0.05 and power: 0.80. About 15% of the patients were assumed to die in breast cancer, and the aim was at a relative risk (RR) of about 1.30. With 2000 patients a detection of a RR of 1.32 would be possible, and even lower for all‐cause mortality. In order to get this number, cases were picked in consecutive order and included in the study if there was an available serum sample attached. In all, 2903 cases were picked to get 2000 patients with a serum sample, all diagnosed 2010 to 2013. Hence, 903 cases were not included due to no available serum (Figure 1).

### 2.3 Clinical Data

Clinical characteristics were obtained from NKBC, which is based on the Swedish Cancer Registry [20]. The record linkage was performed using the unique Swedish identity number. If a patient had duplicate tumours reported in the registry, the tumours had to be more than 90 days apart for the first entry to be used. When there was less than 90 days apart, all entries were manually checked and priority was given to the entry with the most advanced invasive case, according to the TNM stage and/or the entry with complete data. All duplicate cases were checked in order not to use entries including cancer in situ (CIS) tumours, as an individual selected with an invasive tumour may also have contributed with information on CIS. This process enabled the identification of clinical data for the first invasive tumour and ascertained that the blood sample had been taken at the diagnosis of the first invasive cancer, and not in relation to a previous CIS.

Clinical data included tumour characteristics such as type, Nottingham histological grade (NHG), ER, PgR, HER2 and Ki67 expression, size, and lymph node metastases. ER and PgR status were defined as positive if > 10% of nuclei were positive. Lymph node status was defined as positive if present macro‐ or micrometastases. Submicrometastases (isolated tumour cells, ITC, < 0.2 mm) were classified as lymph node negative. HER2 positive tumours were identified with immunohistochemistry (IHC) and for 2+ and 3+ on IHC, completed with in situ hybridization (ISH). Ki67 status was reported by the local pathology department according to their individual cut‐off at the time of examination. Low and intermediary were grouped as low. Age at diagnosis was binned in five‐year intervals. Patients were further divided into groups of < 50, 50–60, 60–70 and > 70 years. Information about menopause status, adjuvant therapy, and surgical procedure was retrieved from the NKBC.

### 2.4 Follow Up

The date of diagnosis was imported from NKBC. All patients received a reference date for diagnosis. For patients without data in the register, the date of diagnosis was set to the date of the tissue sample or else from the date of consent, or from the registration date. Patients were followed from reference date until recurrence (in the analyses using recurrent disease as an endpoint), death or the end of follow‐up. Information on vital status was obtained from The Swedish Population Registry and data on recurrent disease is entered into the NKBC when diagnosed. Recurrent disease was defined as yes or no based on recurrent tumour in the same breast or chest wall, or findings of metastases in axilla, parasternal or supraclavicular. The last date of follow‐up occurred during the period 1 April–31 June 2019. In order to protect the privacy of previous patients, no exact date for the end of follow‐up was given to the authors. The steering committee of SCAN‐B instead provided information on the number of days between the date of diagnosis and end of follow‐up, i.e., the date of recurrent disease, death, or end of follow‐up.

### 2.5 Thyroid Hormone Analysis

Serum samples were collected from patients enrolling in the SCAN‐B study at their treating hospital. Samples were aliquoted and frozen within 2 h. All samples were transported on dry ice to the biobank where they were stored at −80°C. Samples were retrieved in consecutive order to get 2000; however, they were later analysed in a randomised order. Hormone data were analysed at the Department of Clinical Chemistry, Skåne University Hospital. Serum levels of free T3, total T3, free T4 and total T4 were measured using a two‐step electrochemiluminescent immunoassay, ECLI, detection method based on Ruthenium (Ru) derivate and performed on Cobas (NPU03625). The reference ranges and coefficients of variation (CV) were as follows: free T3, 3.6–6.3 pmol/L (CV 5%); total T3, 1.3–3.1 nmol/L (CV 4%); free T4, 12–22 pmol/L (CV 5%); and total T4, 65–180 nmol/L (CV 4%). All in accordance with the manufacturer's instructions and as reported from the laboratory.

### 2.6 Statistical Analysis

The cohort was divided into quartiles (Q) based on levels of free T3, total T3, free T4 and total T4, respectively. The fraction of free T3/free T4 was calculated as an indication of the conversion from T4 to T3, and this ratio was also classified into quartiles.

Descriptive analyses were performed to assess the distribution of established prognostic factors and age among the different thyroid hormone groups and vital status using a chi‐squared test. In addition, hormone quartiles were compared to adjuvant treatment and surgery methods. Missing values in covariates were excluded from the chi‐squared test.

Kaplan–Meier curves were used to compare quartiles of thyroid hormones in relation to all‐cause mortality and recurrent disease. Differences between quartiles were tested using the log‐rank test. Mortality was calculated per 100,000 person‐years and quartiles were compared using Cox proportional hazard analysis to get hazard ratios (HR) and confidence intervals. The proportionality assumption of Cox's model was tested by computing Schoenfeld partial residuals, without detecting any violations (Figure S1). Model one included only the studied thyroid hormone, i.e., it was the crude analysis. Model two was additionally adjusted for age at diagnosis. Model three was adjusted for age, menopause status, diagnosis by screening mammography, size, lymph node status, Nottingham histological grade, histological tumour type, ER status, PgR status and HER2‐receptor status. Ki67 expression was not adjusted for in the analysis due to a large proportion of missing values. Missing values in covariates were coded as a separate category and included in the model. If patients did not have a registered recurrence in the NKBC, or if the follow‐up time was missing in the recurrence‐free interval variable (*n* = 55), these patients were considered to not have had any recurrences, and the total follow‐up time from the date of diagnosis until death or end of follow up was used in the analysis of recurrent disease.

Following the analyses for survival and discovery of a potential threshold effect, total and free T3 analyses were also performed in a post hoc analysis merging Q2 to Q4 into a single group and comparing it to Q1.

Post‐ and premenopausal women may differ regarding a potential association between thyroid hormones and prognosis following breast cancer. The number of events in the premenopausal group was limited and did not allow a meaningful separate analysis; instead, we performed a sensitivity analysis restricted to postmenopausal women regarding the main analyses. The cohort included only 8 men, and the low number did not allow meaningful separate analyses.

SPSS version 27 was used for all statistical analyses, except for the creation of the Shoenfeld residual plot, which was performed using the R software (The R Foundation), version 4.0.4, the level of significance was set to 0.05.

## 3 Results

### 3.1 Descriptive Statistics

Patients with the lowest free T3 levels (Q1) were more likely to be older and postmenopausal (Table 1). Moreover, patients with the highest free T3 levels (Q4) had more often a higher histological grade (Table 1). Total T3 showed a similar pattern as for free T3, but additionally, women with a high total T3 were more likely to have gotten their diagnosis from mammography screening (Table S1). Patients with high levels of free T4 (Q4) were relatively old at baseline, as compared to patients with low levels, and they were less often diagnosed following screening (Table S2). The pattern for total T4 was similar to that seen for free T4 (Table S3). The analysis of fT3/fT4 fraction in patients with breast cancer showed a similar pattern as for the analysis of free T3 and T4 quartiles (Table S4). The distributions of adjuvant therapy and surgical procedures were similar over different quartiles of studied hormones (Tables S5–S9).

**TABLE 1:** Free T3 levels in relation to percentage distribution of tumour characteristics, age and menopausal status.

Factor	Free T3					*p*
	≤ 4.20 pmol/L	4.21–4.56 pmol/L	4.57–4.95 pmol/L	> 4.96 pmol/L	All	
	*n* = 510	*n* = 501	*n* = 490	*n* = 499	*n* = 2000	
Age at diagnosis						
< 50	4.9	10.0	10.4	10.6	9.0	< 0.001
50–60	22.0	30.1	32.4	37.7	30.5	
60–70	36.1	31.9	39.0	33.3	35.1	
> 70	37.1	27.9	18.2	18.2	25.5	
Menopausal status						
Premenopausal	12.5	18.6	20.6	21.6	18.3	0.005
Postmenopausal	82.5	75.8	75.1	71.3	76.3	
Uncertain	3.3	4.4	3.7	5.4	4.2	
Missing	1.6	1.2	0.6	1.6	1.3	
Diagnosis by screening mammography						
No	51.0	46.7	42.2	45.5	46.4	0.198
Yes	48.0	52.3	56.1	53.3	52.4	
Missing	1.0	1.0	1.6	1.2	1.2	
Tumour size						
≤ 20 mm	66.5	71.7	70.2	63.9	68.1	0.191
> 20 mm	32.7	27.5	29.0	35.3	31.2	
Tumour type (histological)						
Other + mixed	8.2	7.0	6.1	6.0	6.9	0.205
Ductal	79.4	76.6	81.4	82.7	80.0	
Lobular	12.4	16.0	12.4	11.2	13.0	
Lymph node status						
Node negative	65.5	65.7	63.1	62.5	64.2	0.9
Node positive	30.8	30.9	32.4	33.3	31.9	
Missing	3.7	3.4	4.5	4.2	4.0	
Nottingham histological grade						
Grade 1	19.8	17.8	23.9	15.2	19.2	0.01
Grade 2	46.5	48.1	45.9	43.7	46.1	
Grade 3	30.8	31.3	27.1	38.3	31.9	
Missing	2.9	2.8	3.1	2.8	2.9	
Oestrogen receptor status						
Negative	14.3	12.6	13.3	16.0	14.1	0.399
Positive	85.7	87.0	86.5	83.4	85.7	
Progesterone receptor status						
Negative	28.4	26.9	26.3	39.9	27.9	0.504
Positive	71.6	72.9	73.3	69.5	71.8	
HER2 status						
Negative	87.6	85.0	85.1	87.0	86.2	0.32
Positive	11.6	13.8	12.4	11.8	12.4	
Missing	0.8	1.2	2.4	1.2	1.4	
Ki67 status						
Low	11.6	10.4	12.0	11.4	11.4	0.713
High	12.9	13.8	10.4	13.6	12.7	
Missing	75.5	75.8	77.6	74.9	75.9	

*Note:* All data is presented as column percentage. Missing values not presented if < 1%.

Patients who died during follow‐up were older at diagnosis, more often post‐menopausal, had larger tumours, more involved lymph nodes, more often higher NHG, and ER‐, PgR‐ negative tumours. Higher Ki67 expression was found for these patients, and they were less likely to have been diagnosed by screening mammography (Table S10).

### 3.2 Non‐Included Patients Compared to Included

The main reason not to include a patient in the consecutive series was the lack of available blood samples. Among non‐included patients, there was a slightly higher proportion of diagnosis from mammography screening (61.2%), as compared to included patients (53%) (Table S11). Other factors were similar in these groups. The same was seen in relation to adjuvant therapy and surgical procedures (Table S12).

### 3.3 Survival of Breast Cancer Patients

**FIGURE 2:**
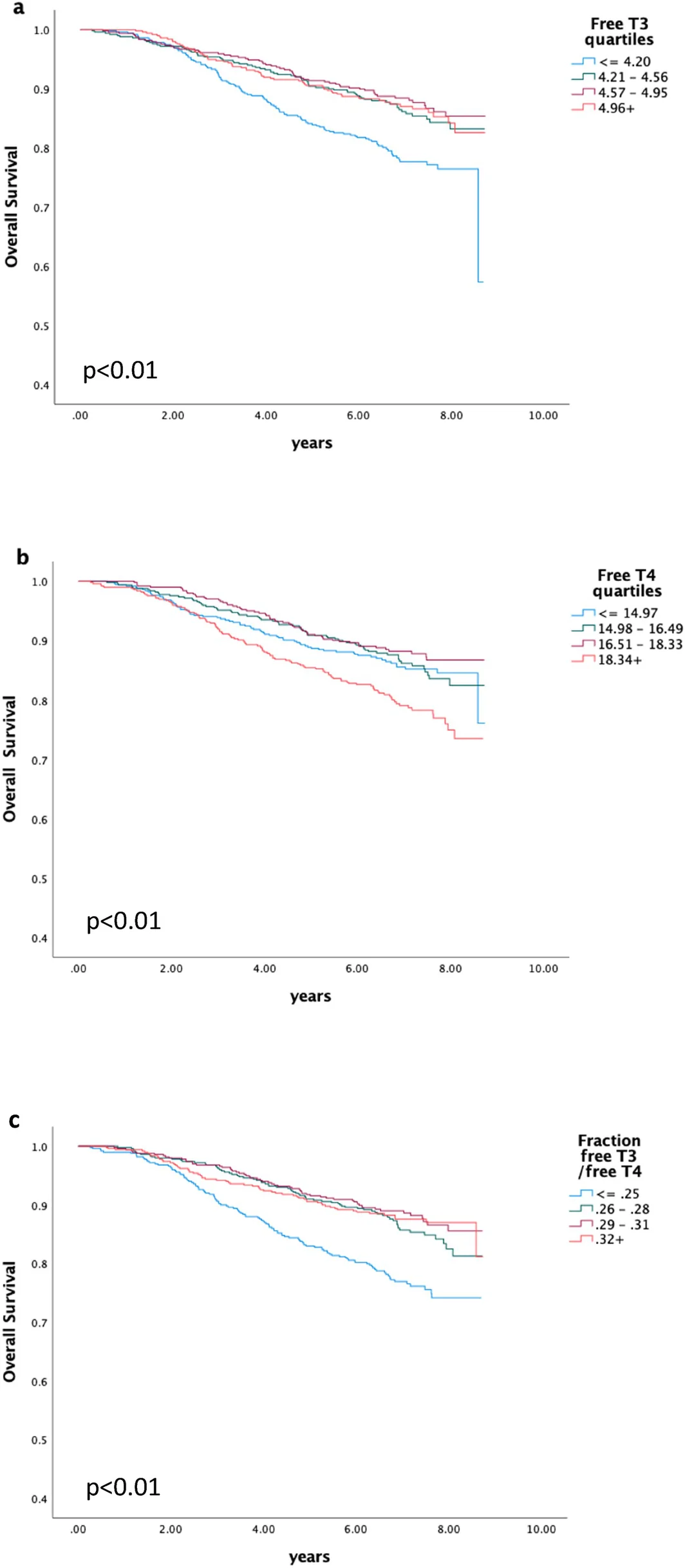
Free T3 (a), free T4 (b) and the ratio free T3/T4 (c) in relation to survival following a breast cancer diagnosis, using all‐cause mortality as endpoint.

**FIGURE 3:**
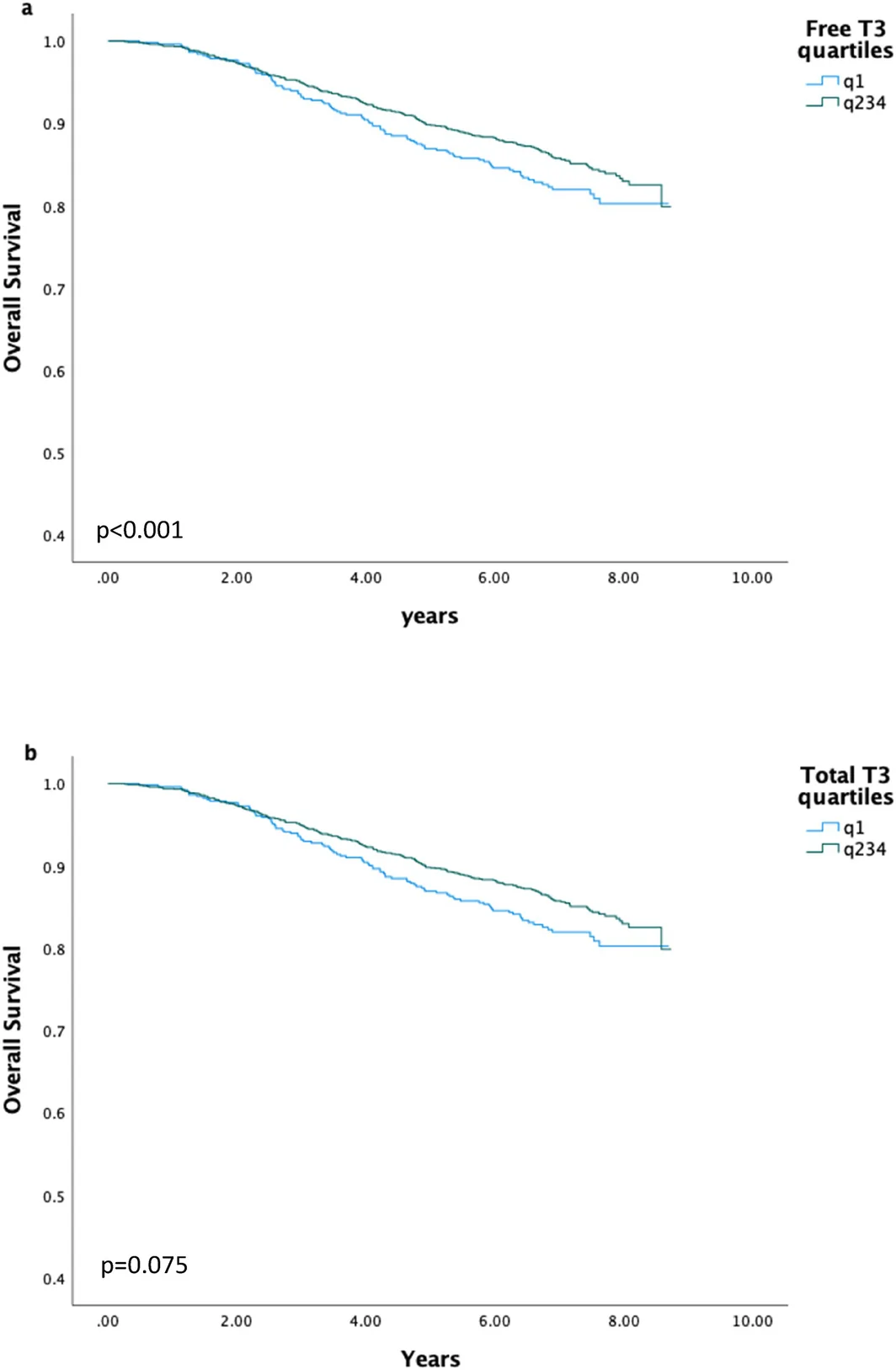
Post hoc analysis of free T3 (a) and Total T3 (b) in relation to survival following a breast cancer diagnosis, using all‐cause mortality as endpoint.

During the follow‐up period, 310 deaths were registered among breast cancer patients in the cohort. All‐cause mortality related to free T3 was, as compared to Q1, lower in Q2 (HR; 0.62: 0.46–0.83), Q3 (0.54: 0.39–0.73) and Q4 (0.60: 0.44–0.81) (Table 2 and Figure 2a). In the age‐adjusted model, the association was still moderately strong for Q2 and Q3, but not for Q4. In the fully adjusted model, only Q2 showed a statistically significant HR: 0.67 (0.51–0.96). In the post hoc analysis of free T3, mortality was lower in the merged group consisting of Q2, Q3 and Q4 compared to Q1, *p* < 0.001 (Figure 3a). The corresponding HR for the merged group compared to Q1 was 0.58 (0.46–0.74). Slightly attenuated in the adjusted analysis, but still statistically significant (Table 3). Considering total T3, the mortality was similar in different quartiles, *p* = 0.348 (Figure S2a), but the crude analysis indicated a somewhat lower mortality in Q2–4 as compared to Q1 (Table 2). This was confirmed in the post hoc analysis, but no associations related to total T3 were statistically significant (Table 3, Figure 3b).

**TABLE 2:** Thyroid hormones and all‐cause mortality.

Hormone	Quartile	Patients (*n*)	Dead (*n*)	Mortality/100 person years	HR^a^	HR^b^	HR^c^
Free T3	1	510	111	3.37	1	1	1
	2	501	71	2.10	0.62 (0.46–0.83)	0.72 (0.53–0.97)	0.75 (0.55–1.02)
	3	490	61	1.83	0.54 (0.39–0.73)	0.71 (0.52–0.97)	0.67 (0.51–0.96)
	4	498	67	2.02	0.60 (0.44–0.81)	0.82 (0.61–1.12)	0.75 (0.55–1.03)
Total T3	1	513	92	2.72	1	1	1
	2	511	75	2.17	0.79 (0.58–1.07)	0.87 (0.64–1.18)	0.88 (0.65–1.20)
	3	493	74	2.27	0.83 (0.61–1.13)	1.05 (0.77–1.43)	0.98 (0.72–1.34)
	4	482	69	2.14	0.78 (0.57–1.07)	1.04 (0.76–1.43)	0.92 (0.67–1.27)
Free T4	1	505	73	2.16	1	1	1
	2	495	70	2.11	0.98 (0.70–1.36)	0.96 (0.69–1.34)	0.97 (0.70–1.35)
	3	503	61	1.78	0.82 (0.59–1.16)	0.75 (0.54–1.06)	0.71 (0.50–1.00)
	4	496	106	3.30	1.53 (1.14–2.07)	1.31 (0.97–1.77)	1.25 (0.93–1.70)
Total T4	1	500	67	2.00	1	1	1
	2	500	67	1.98	0.99 (0.71–1.39)	0.90 (0.64–1.26)	0.91 (0.65–1.29)
	3	504	72	2.14	1.07 (0.77–1.50)	0.97 (0.69–1.35)	0.92 (0.66–1.28)
	4	495	104	3.24	1.63 (1.20–2.22)	1.38 (1.01–1.88)	1.24 (0.91–1.69)
Free T3/free T4	1	500	116	3.64	1	1	1
	2	500	72	2.11	0.57 (0.43–0.77)	0.65 (0.48–0.87)	0.65 (0.48–0.87)
	3	500	60	1.78	0.48 (0.35–0.66)	0.64 (0.47–0.88)	0.65 (0.47–0.89)
	4	500	62	1.85	0.50 (0.37–0.68)	0.70 (0.51–0.95)	0.71 (0.52–0.97)

^a^ Crude analysis.

^b^ Adjusted for age.

^c^ Adjusted for age, menopause status, tumour size, diagnosis by screening mammography, oestrogen receptor status, progesterone receptor status, HER2‐receptor status, Nottingham histological grade, lymph node status and histological tumour type.

**TABLE 3:** Post hoc analysis of free T3 and total T3 in relation to all‐cause mortality.

Hormone	Quartile	Patients (*n*)	Dead (*n*)	Mortality/100 person years	HR^a^	HR^b^	HR^c^
Free T3	1	510	111	3.37	1	1	1
	2–4	1489	199	1.98	0.58 (0.46–0.74)	0.75 (0.59–0.95)	0.73 (0.58–0.93)
Total T3	1	513	92	2.72	1	1	1
	2–4	1486	218	2.19	0.80 (0.63–1.02)	0.98 (0.76–1.25)	0.92 (0.72–1.19)

^a^ Crude analysis.

^b^ Adjusted for age.

^c^ Adjusted for age, menopause status, tumour size, diagnosis by screening mammography, oestrogen receptor status, progesterone receptor status, HER2‐receptor status, Nottingham histological grade, lymph node status and histological tumour type.

High levels of free T4 showed a high mortality rate, *p* < 0.001 (Figure 2b). The all‐cause mortality for Q4 was high compared to Q1 (1.53: 1.14–2.07) (Table 2). The results were, however, attenuated when adjusted for age (1.31: 0.97–1.77) and other covariates (1.25: 0.93–1.70) (Table 2). Total T4 had a high mortality rate (Figure S2b). Total T4 showed a strong association with a high all‐cause mortality for Q4 vs. Q1 in the crude analysis (1.63: 1.20–2.22), and when adjusted for age (1.38: 1.01–1.88), although this association was weaker when the analysis was adjusted for all covariates (1.24: 0.91–1.69) (Table 2).

A high ratio of free T3/T4 was associated with a low all‐cause mortality (Figure 2c). This was seen for all quartiles, and also following adjustments; Q2 (0.65: 0.48–0.87), Q3 (0.65: 0.47–0.89), Q4 (0.71: 0.52–0.97) (Table 2).

Following the sensitivity analysis restricted to postmenopausal women, all main results were very similar (Table S13), as compared to corresponding HRs in the total cohort (Table 2).

### 3.4 Recurrence in Breast Cancer

There were 167 patients with recurrent disease among 2000 patients. No association was found between thyroid hormone levels and recurrence in breast cancer. Crude and adjusted analyses were very similar (Figure S3 and Table S14). For the fraction of freeT3/T4 there was a lower risk of recurrence for patients in Q2 compared to Q1 (0.63: 0.40–0.98), but no such difference was not seen for Q3 or Q4.

## 4 Discussion

The aim of this study was to investigate the prognostic value of thyroid hormones in breast cancer patients. We found a threshold effect for free T3 levels in breast cancer patients, where intermediate and high levels were associated with a low mortality, i.e., a favourable prognosis. This was further supported in the analyses of the free T3/T4 ratio. The crude analysis for free and total T4 indicated, if anything, a higher mortality for the highest quartiles, but these associations did not remain following adjustments for age and other established prognostic factors.

Previous studies have found a positive association between high pre‐diagnostic total T3 and a high breast cancer‐specific mortality [5]. In the same study, there was no association between total T3 and all‐cause mortality. Brandt et al. found higher pre‐diagnostic levels of T4 to be associated with lower breast cancer mortality [1]. In our study, we found no such indication; on the contrary, there seemed to be a tendency towards higher mortality for patients with high T4 levels.

In a small study of thyroid hormones and the Silencing Mediator of Retinoid and Thyroid receptor (SMRT) expression in breast cancer patients, compared to benign breast disease, there was an association of low levels of free T3, high levels of free T4, and a higher expression of SMRT in breast cancer. Furthermore, elevated SMRT was associated with negative prognostic factors [2]. Our study is in line with these findings and may suggest that there is a difference between pre‐ and post‐diagnostically measured thyroid hormones and their relationship to breast cancer prognosis. DIO1 is crucial for conversion of T4 to T3 and interestingly, Demircan et al. found DIO1mRNA expression to be associated with improved prognosis for breast cancer if selenium levels are high. In the present study, the fraction of free T3/T4 is an indicator of the level of conversion of thyroid hormones, i.e., a higher fraction implies a higher DIO1 activity. The results from our study are in line with this and showed lower mortality for quartiles with higher free T3 fraction.

One aspect of the difference between our results and the conflicting results from other studies might lay in the time of blood samples drawn from the patient. In patients where a breast cancer is present in the body, cancer may have interfered with thyroid levels. Previous studies on free T3 levels in breast cancer have found both significantly higher and lower levels of T3 compared to controls [21, 22]. It would probably be more relevant for the physician to know the prognostic value of thyroid status at cancer diagnosis rather than before. Partly because the probability of a breast cancer patient having a pre‐diagnostic thyroid sample is low, and partly because cancer might have changed the thyroid status.

In individuals without thyroid dysfunction, measurement of TSH and free T4 has shown to be most accurately reflecting thyroid function. T3 is due to its short half‐life (24 h) and instability for homeostatic changes, not as representative [23]. However, the long‐term individual range of thyroid hormones is stable, although the inter‐individual changes can be large [24]. Studies have shown that the normal reference in laboratory tests can be of pathological value to some individuals due to the large variation between normal set points [24]. In the present study, laboratory tests of thyroid hormones showed stable results with a low coefficient of variation for reference values. With this knowledge about individual changes in thyroid analyte levels, it would be considered enough with just one blood sample, as in the present study, although there would be a value in testing for TSH as well and the lack of data on TSH is a limitation.

There were no strong associations or clear patterns related to hormonal levels and recurrent disease. Most HR were close to unity, but a single category related to the free T3/T4 fraction had a low risk of recurrent disease. However, this should be regarded with caution given the possibilities of a chance finding. Another aspect is related to the validity of information on recurrent disease. While cause of death is mandatory to report to the Swedish Population Register, reporting recurrence to the NKBC, from where our recurrence data comes from, is not mandatory by law, and relies on health care personnel reporting. Therefore, this data might not be as complete as the data on mortality, and underreporting of recurrent disease may have been related to varied routines over time and between health care institutions. In this study, there were 167 reported recurrent diseases. To compare with a previous observational study on 8062 women in the Netherlands with invasive, non‐metastatic breast cancer who measured recurrence rate and reported 0.02049 recurrent diseases per person‐year [25]. In the present study, the same number was 0.01279 recurrences per person‐year. This implies that there was some under‐reporting of recurrent disease in our study. However, there is nothing to suggest that such under‐reporting would have been associated with the analysed biomarkers or specific thyroid conditions. The main effect of such underreporting would, hence, be a poor statistical power.

A potential association between thyroid hormones and prognosis may be different according to menopausal status. This was, however, not indicated in the sensitivity analysis. Furthermore, in the present study, adjustments were made for established prognostic factors, including menopausal status. Although there was a large amount of missing data for Ki67, the data for grade, which is strongly correlated with Ki67, was almost complete. Since thyroid function and breast cancer prognosis may be affected by several other environmental factors, such as smoking, alcohol consumption, and overweight, it would have been interesting to add these factors if they had been available, and the lack of this information is a study limitation. Furthermore, it was a limitation that the small number of men did not allow a separate analysis.

Patients in this study were not excluded from the analysis based on previous thyroid conditions as this was not known. In hypothyroidism, the primary indicator is TSH, and initially, T4 and T3 are within the normal range [26]. If some of our patients were undiagnosed with hypothyroidism, or treated with L‐thyroxine, it is not possible to know based on our data. Nevertheless, the individual value of the hormones apart from the function of the thyroid gland can still be validated as a prognostic factor for breast cancer. In order to consider the association between clinically defined conditions, TSH would have been necessary, and, as mentioned above, the lack of this data is a limitation of the present study.

In the euthyroid sick syndrome, critical illness or systemic inflammation contributes to low free T3 levels, initially higher T4 levels, and is a prognostic marker for mortality in various systemic diseases and cancers [27, 28]. It would be possible to suggest that the higher rate of all‐cause mortality in the lowest quartile for free T3 in the present study could reflect patients with higher inflammatory burden and comorbidity. If caused by the same mechanisms as euthyroid sick syndrome, although not as prominent, this would lead to less transformation of free T4 to free T3 in peripheral blood.

An additional methodological issue concerns the effect of treatment on thyroid hormone levels, as it has been reported that they may be affected by both adjuvant chemotherapy and adjuvant antihormonal therapy [29, 30]. The current study obtained blood samples at diagnosis, and hence, surgical or medical treatment per se could not have affected thyroid hormonal levels.

To our knowledge, this is the largest study of thyroid function on breast cancer mortality. Measuring thyroid levels after breast cancer prognosis is approaching a more clinical aspect of the interpretation of thyroid function in breast cancer outcome.

Many patients in the cohort are adding to the strengths of this study. The SCAN‐B study has a high participating level and a low drop‐out [20]. This has made SCAN‐B a comprehensive and well‐represented database of the population in the south of Sweden.

In conclusion, the present study included 2000 newly diagnosed breast cancer patients for thyroid level analysis. The results showed that patients with a relatively high free T3 at the time of diagnosis have a lower all‐cause mortality and that T3 levels may be a potential prognostic marker for breast cancer, but further studies need to approach the relevance of these findings.

## Author Contributions

**Martin Malmberg:** conceptualization. **Ylva Heyman:** conceptualization. **Annie Brange:** conceptualization. **Johan Vallon‐Christersson:** conceptualization. **Lisa Rydén:** conceptualization. **Lao H. Saal:** conceptualization. **Åke Borg:** conceptualization. **Lutz Schomburg:** conceptualization. **Kamil Demircan:** conceptualization. **Jonas Manjer:** conceptualization.

## Funding

Funding was received from the Swedish Foundation for Strategic Research, Swedish Research Council, Swedish Cancer Society, The Mrs. Berta Kamprad Foundation, Knut and Alice Wallenberg Foundation, VINNOVA, Governmental Funding of Clinical Research within National Health Service, Lund University Medical Faculty Foundation, Skåne University Hospital Foundation, and Malmö University Hospital Cancer Foundation.

## Ethics Statement

The study was approved by the Regional Ethical Review Board of Lund in 2007 and 2009 (diary numbers 2007/155, 2009/658, 2009/659,62014/8), the county governmental biobank centre and the Swedish Data Inspection group (diary number 364–2010).

## Consent

All participants signed an informed consent.

## Conflicts of Interest

The authors declare no conflicts of interest.

## Supporting information


**Figure S1:** Selected Schoenfeld residual plots for checking the proportional hazards assumption. The plots indicate that no violations of the proportional hazards assumption were observed.

**Figure S2:** Total T3 (a) and Total T4 (b) in relation to survival following a breast cancer diagnosis, using all‐cause mortality as endpoint.

**Figure S3:** Thyroid hormones in relation to risk of recurrent disease.

**Table S1:** Total T3 levels in relation to percentage distribution of tumour characteristics, age and menopausal status.
**Table S2:** Free T4 levels in relation to percentage distribution of tumour characteristics, age and menopausal status.
**Table S3:** Total T4 levels in relation to percentage distribution of tumour characteristics, age and menopausal status.
**Table S4:** Free T3/T4 ratio levels in relation to percentage distribution of tumour characteristics, age and menopausal status.
**Table S5:** Free T3 levels in relation percentage distribution of adjuvant therapy and surgical procedure.
**Table S6:** Total T3 levels in relation percentage distribution of adjuvant therapy and surgical procedure.
**Table S7:** Free T4 levels in relation percentage distribution of adjuvant therapy and surgical procedure.
**Table S8:** Total T4 levels in relation percentage distribution of adjuvant therapy and surgical procedure.
**Table S9:** Free T3/T4 levels in relation percentage distribution of adjuvant therapy and surgical procedure.
**Table S10:** Vital status at end of follow up in relation to percentage distribution of tumour characteristics, age and menopausal status.
**Table S11:** Non‐participants as compared to participants in relation to percentage distribution of tumour characteristics, age and menopausal status.
**Table S12:** Non‐participants as compared to participants in relation to percentage distribution of adjuvant therapy and surgical procedure.
**Table S13:** Thyroid hormones and all‐cause mortality in postmenopausal patients.
**Table S14:** Thyroid hormones and recurrent disease.

## Data Availability

The data that support the findings of this study are available on request from the corresponding author. The data are not publicly available due to privacy or ethical restrictions.

## References

[1] J. Brandt , S. Borgquist , M. Almquist , and J. Manjer , “Thyroid Function and Survival Following Breast Cancer,” British Journal of Surgery 103, no. 12 (2016): 1649–1657. 27599301 10.1002/bjs.10284

[2] B. Nisman , T. M. Allweis , E. Carmon , et al., “Thyroid Hormones, Silencing Mediator for Retinoid and Thyroid Receptors and Prognosis in Primary Breast Cancer,” Anticancer Research 40, no. 11 (2020): 6417–6428. 33109580 10.21873/anticanres.14663

[3] I. Muller , C. Giani , L. Zhang , et al., “Does Thyroid Peroxidase Provide an Antigenic Link Between Thyroid Autoimmunity and Breast Cancer?,” International Journal of Cancer 134, no. 7 (2014): 1706–1714. 24114667 10.1002/ijc.28493

[4] J. Jiskra , J. Barkmanova , Z. Limanova , et al., “Thyroid Autoimmunity Occurs More Frequently in Women With Breast Cancer Compared to Women With Colorectal Cancer and Controls but It Has no Impact on Relapse‐Free and Overall Survival,” Oncology Reports 18, no. 6 (2007): 1603–1611. 17982651

[5] A. Tosovic , A. G. Bondeson , L. Bondeson , U. B. Ericsson , and J. Manjer , “Triiodothyronine Levels in Relation to Mortality From Breast Cancer and All Causes: A Population‐Based Prospective Cohort Study,” European Journal of Endocrinology 168, no. 4 (2013): 483–490. 23258272 10.1530/EJE-12-0564

[6] C. W. Elston and I. O. Ellis , “Pathological Prognostic Factors in Breast Cancer. I. The Value of Histological Grade in Breast Cancer: Experience From a Large Study With Long‐Term Follow‐Up,” Histopathology 19, no. 5 (1991): 403–410. 1757079 10.1111/j.1365-2559.1991.tb00229.x

[7] M. Cianfrocca and L. J. Goldstein , “Prognostic and Predictive Factors in Early‐Stage Breast Cancer,” Oncologist 9, no. 6 (2004): 606–616. 15561805 10.1634/theoncologist.9-6-606

[8] L. Rydén , M. Haglund , P. O. Bendahl , et al., “Reproducibility of Human Epidermal Growth Factor Receptor 2 Analysis in Primary Breast Cancer: A National Survey Performed at Pathology Departments in Sweden,” Acta Oncologica 48, no. 6 (2009): 860–866. 19353340 10.1080/02841860902862511

[9] S. Dinda , A. Sanchez , and V. Moudgil , “Estrogen‐Like Effects of Thyroid Hormone on the Regulation of Tumor Suppressor Proteins, p53 and Retinoblastoma, in Breast Cancer Cells,” Oncogene 21, no. 5 (2002): 761–768. 11850804 10.1038/sj.onc.1205136

[10] L. C. Hall , E. P. Salazar , S. R. Kane , and N. Liu , “Effects of Thyroid Hormones on Human Breast Cancer Cell Proliferation,” Journal of Steroid Biochemistry and Molecular Biology 109, no. 1 (2008): 57–66. 18328691 10.1016/j.jsbmb.2007.12.008

[11] J. M. Silva , G. Domínguez , J. M. González‐Sancho , et al., “Expression of Thyroid Hormone Receptor/erbA Genes Is Altered in Human Breast Cancer,” Oncogene 21, no. 27 (2002): 4307–4316. 12082618 10.1038/sj.onc.1205534

[12] Z. Li , Z. H. Meng , R. Chandrasekaran , et al., “Biallelic Inactivation of the Thyroid Hormone Receptor β1 Gene in Early Stage Breast Cancer,” Cancer Research 62, no. 7 (2002): 1939. 11929806

[13] M. M. França , A. German , G. W. Fernandes , et al., “Human Type 1 Iodothyronine Deiodinase (DIO1) Mutations Cause Abnormal Thyroid Hormone Metabolism,” Thyroid 31, no. 2 (2021): 202–207. 32718224 10.1089/thy.2020.0253 PMC7891200

[14] K. Demircan , Y. Bengtsson , T. S. Chillon , et al., “Matched Analysis of Circulating Selenium With the Breast Cancer Selenotranscriptome: A Multicentre Prospective Study,” Journal of Translational Medicine 21, no. 1 (2023): 658. 37741974 10.1186/s12967-023-04502-y PMC10517476

[15] J. Brandt , S. Borgquist , and J. Manjer , “Prospectively Measured Thyroid Hormones and Thyroid Peroxidase Antibodies in Relation to Risk of Different Breast Cancer Subgroups: A Malmö Diet and Cancer Study,” Cancer Causes & Control 26, no. 8 (2015): 1093–1104. 26033776 10.1007/s10552-015-0602-8

[16] A. Tosovic , A. G. Bondeson , L. Bondeson , U. B. Ericsson , and J. Manjer , “T3 Levels in Relation to Prognostic Factors in Breast Cancer: A Population‐Based Prospective Cohort Study,” BMC Cancer 14 (2014): 536. 25060772 10.1186/1471-2407-14-536 PMC4131035

[17] M. Søgaard , D. K. Farkas , V. Ehrenstein , J. O. Jørgensen , O. M. Dekkers , and H. T. Sørensen , “Hypothyroidism and Hyperthyroidism and Breast Cancer Risk: A Nationwide Cohort Study,” European Journal of Endocrinology 174, no. 4 (2016): 409–414. 26863886 10.1530/EJE-15-0989

[18] R. S. Wahdan‐Alaswad , S. M. Edgerton , H. Salem , et al., “Exogenous Thyroid Hormone Is Associated With Shortened Survival and Upregulation of High‐Risk Gene Expression Profiles in Steroid Receptor‐Positive Breast Cancers,” Clinical Cancer Research 27, no. 2 (2021): 585–597. 33097494 10.1158/1078-0432.CCR-20-2647

[19] L. H. Saal , J. Vallon‐Christersson , J. Häkkinen , et al., “The Sweden Cancerome Analysis Network—Breast (SCAN‐B) Initiative: A Large‐Scale Multicenter Infrastructure Towards Implementation of Breast Cancer Genomic Analyses in the Clinical Routine,” Genome Medicine 7, no. 1 (2015): 20. 25722745 10.1186/s13073-015-0131-9 PMC4341872

[20] L. Rydén , N. Loman , C. Larsson , et al., “Minimizing Inequality in Access to Precision Medicine in Breast Cancer by Real‐Time Population‐Based Molecular Analysis in the SCAN‐B Initiative,” British Journal of Surgery 105, no. 2 (2018): e158–e168. 29341157 10.1002/bjs.10741 PMC5817401

[21] N. Ditsch , S. Liebhardt , F. von Koch , et al., “Thyroid Function in Breast Cancer Patients,” Anticancer Research 30, no. 5 (2010): 1713–1717. 20592366

[22] D. P. Rose and T. E. Davis , “Plasma Triiodothyronine Concentrations in Breast Cancer,” Cancer 43, no. 4 (1979): 1434–1438. 445341 10.1002/1097-0142(197904)43:4<1434::aid-cncr2820430433>3.0.co;2-s

[23] H. Li , X. Yuan , L. Liu , et al., “Clinical Evaluation of Various Thyroid Hormones on Thyroid Function,” International Journal of Endocrinology 2014 (2014): 618572. 25548564 10.1155/2014/618572 PMC4274666

[24] S. Andersen , K. M. Pedersen , N. H. Bruun , and P. Laurberg , “Narrow Individual Variations in Serum T(4) and T(3) in Normal Subjects: A Clue to the Understanding of Subclinical Thyroid Disease,” Journal of Clinical Endocrinology and Metabolism 87, no. 3 (2002): 1068–1072. 11889165 10.1210/jcem.87.3.8165

[25] M. C. van Maaren , L. de Munck , L. J. Strobbe , et al., “Ten‐Year Recurrence Rates for Breast Cancer Subtypes in The Netherlands: A Large Population‐Based Study,” International Journal of Cancer 144, no. 2 (2019): 263–272. 30368776 10.1002/ijc.31914

[26] P. Nilsson‐Ehle , M. B. Söderlund , E. Theodorsson , C. Becker , and C. Laurell , Laurells Klinisk Kemi i praktisk medicin, 9th ed. (Studentlitteratur, 2012).

[27] A. Moura Neto and D. E. Zantut‐Wittmann , “Abnormalities of Thyroid Hormone Metabolism During Systemic Illness: The Low T3 Syndrome in Different Clinical Settings,” International Journal of Endocrinology 2016 (2016): 2157583. 27803712 10.1155/2016/2157583 PMC5075641

[28] R. Gao , J. H. Liang , L. Wang , et al., “Low T3 Syndrome Is a Strong Prognostic Predictor in Diffuse Large B Cell Lymphoma,” British Journal of Haematology 177, no. 1 (2017): 95–105. 28146267 10.1111/bjh.14528

[29] D. Marina , K. Buch‐Larsen , L. Gillberg , et al., “Chemotherapy for Post‐Menopausal Women With Early Breast Cancer Seems Not to Result in Clinically Significant Changes in Thyroid Function,” Cancer Medicine 13, no. 15 (2024): e70015. 39108148 10.1002/cam4.70015 PMC11303825

[30] D. Marina , A. Nielsen , K. Buch‐Larsen , M. Andersson , Å. K. Rasmussen , and P. Schwarz , “Impact of Aromatase Inhibitors on Thyroid Function in Postmenopausal Women With Early‐Stage Breast Cancer: A Prospective Controlled Study,” Cancer Treat Res Commun 45 (2025): 101020. 41172892 10.1016/j.ctarc.2025.101020

