# Supplementary material for: Serum Levels of Thyroid Hormones in Relation to Breast Cancer Prognosis: A Multicentre Cohort Study
Source: Cancer Med. 2026 Sep 9;15(9):e72264. doi: 10.1002/cam4.72264 (PMC13559021; doi:10.1002/cam4.72264)
Supplement: Supplementary file 2 [file CAM4-15-e72264-s002.docx]

**Table S1.** Total T3 levels in relation to percentage distribution of tumour characteristics, age, and menopausal status.

| Factor | | Total T3 | | | | |  |
| --- | --- | --- | --- | --- | --- | --- | --- |
|  | | ≤1.36 pmol/L | 1.36 - 1.53 pmol/L | 1.53 - 1.72 pmol/L | >1.72 pmol/L | All | p-value |
|  | | n=513 | n=511 | n=494 | n=482 | n=2000 |  |
| Age at diagnosis | <50 | 7.2 | 8.2 | 8.5 | 12.0 | 9.0 | <0.001 |
|  | 50-60 | 23.0 | 28.2 | 36.0 | 35.3 | 30.5 |  |
|  | 60-70 | 35.5 | 35.4 | 33.4 | 35.9 | 35.1 |  |
|  | >70 | 34.3 | 28.2 | 21.9 | 16.8 | 25.5 |  |
| Menopausal status | Premenopausal | 15.8 | 15.7 | 20.2 | 21.8 | 18.3 | 0.044 |
|  | Postmenopausal | 78.8 | 79.1 | 75.3 | 71.6 | 76.3 |  |
|  | Uncertain | 3.7 | 4.3 | 3.0 | 5.8 | 4.2 |  |
|  | Missing | 1.8 | 1.0 | 1.4 | 0.8 | 1.3 |  |
| Diagnosis by screening mammography | No | 52.0 | 46.0 | 45.3 | 41.9 | 46.4 | 0.037 |
|  | Yes | 46.8 | 52.4 | 53.2 | 57.5 | 52.4 |  |
|  | Missing | 1.2 | 1.6 | 1.4 | 0.6 | 1.2 |  |
| Tumour size | ≤20mm | 69.6 | 69.7 | 67.2 | 65.6 | 68.1 | 0.534 |
|  | >20mm | 29.6 | 29.2 | 32.0 | 34.0 | 31.2 |  |
| Tumour type (histological) | Other + mixed | 7.6 | 7.4 | 7.5 | 4.8 | 6.9 | 0.062 |
|  | Ductal | 79.3 | 78.7 | 78.1 | 84.0 | 80.1 |  |
|  | Lobular | 13.1 | 13.9 | 13.8 | 11.2 | 13.0 |  |
| Lymph node status | Node negative | 65.1 | 62.4 | 64.0 | 65.4 | 64.2 | 0.812 |
|  | Node positive | 31.6 | 33.9 | 31.2 | 30.7 | 31.9 |  |
|  | Missing | 2.1 | 1.8 | 4.0 | 3.7 | 2.9 |  |
| Nottingham histological grade | Grade 1 | 18.7 | 19.8 | 19.8 | 18.3 | 19.2 | 0.184 |
|  | Grade 2 | 49.5 | 47.6 | 43.7 | 43.2 | 46.1 |  |
|  | Grade 3 | 29.6 | 30.9 | 32.4 | 34.9 | 31.9 |  |
|  | Missing | 2.1 | 1.8 | 4.0 | 3.7 | 2.9 |  |
| Estrogen receptor status | Negative | 12.7 | 12.7 | 15.4 | 15.6 | 14.1 | 0.540 |
|  | Positive | 87.1 | 86.7 | 84.4 | 84.2 | 85.7 |  |
| Progesterone receptor status | Negative | 26.5 | 26.4 | 30.0 | 28.8 | 27.9 | 0.660 |
|  | Positive | 73.4 | 73.4 | 70.0 | 71.1 | 72.0 |  |
| HER2 status | Negative | 86.4 | 87.5 | 85.0 | 85.9 | 86.2 | 0.875 |
|  | Positive | 12.3 | 11.5 | 13.6 | 13.3 | 12.7 |  |
|  | Missing | 1.4 | 1.0 | 1.4 | 1.9 | 1.4 |  |
| Ki67 status | Low | 10.3 | 9.6 | 11.5 | 14.1 | 11.4 | 0.287 |
|  | High | 12.3 | 11.7 | 13.6 | 13.3 | 12.7 |  |
|  | Missing | 77.4 | 78.7 | 74.9 | 72.6 | 75.9 |  |

All data is presented as column percentage. Missing values not presented if <1%.

**Table S2.** Free T4 levels in relation to percentage distribution of tumour characteristics, age, and menopausal status.

| Factor | | Free T4 | | | | |  |
| --- | --- | --- | --- | --- | --- | --- | --- |
|  | | ≤14.97 pmol/L | 14.98 - 16.49 pmol/L | 16.51 - 18.33 pmol/L | >18.34 pmol/L | All | p-value |
|  | | n=505 | n=495 | n=503 | n=497 | n=2000 |  |
| Age at diagnosis | <50 | 9.7 | 10.3 | 8.5 | 7.2 | 9.0 | 0.003 |
|  | 50-60 | 34.3 | 30.9 | 31.8 | 24.9 | 30.5 |  |
|  | 60-70 | 34.1 | 37.6 | 32.6 | 36.0 | 35.1 |  |
|  | >70 | 22.0 | 21.2 | 27.0 | 31.6 | 25.5 |  |
| Menopausal status | Premenopausal | 19.0 | 20.4 | 17.1 | 16.7 | 18.3 | 0.335 |
|  | Postmenopausal | 74.1 | 75.2 | 76.5 | 79.3 | 76.3 |  |
|  | Uncertain | 5.5 | 3.2 | 5.2 | 2.8 | 4.2 |  |
|  | Missing | 1.4 | 1.2 | 1.2 | 1.2 | 1.3 |  |
| Diagnosis by screening mammography | No | 41.2 | 46.9 | 47.9 | 49.7 | 46.4 | 0.039 |
|  | Yes | 58.0 | 51.9 | 51.3 | 48.3 | 52.4 |  |
|  | Missing | 0.8 | 1.2 | 0.8 | 2.0 | 1.2 |  |
| Tumour size | ≤20mm | 68.9 | 71.1 | 68.6 | 63.6 | 68.1 | 0.064 |
|  | >20mm | 29.7 | 28.5 | 30.4 | 36.0 | 31.2 |  |
| Tumour type (histological) | Other + mixed | 8.1 | 6.1 | 5.8 | 7.4 | 6.9 | 0.889 |
|  | Ductal | 79.2 | 80.0 | 80.9 | 79.9 | 80.0 |  |
|  | Lobular | 12.5 | 13.7 | 13.3 | 12.5 | 13.0 |  |
| Lymph node status | Node negative | 62.8 | 65.7 | 68.2 | 60.2 | 64.2 | 0.122 |
|  | Node positive | 33.5 | 29.7 | 28.0 | 36.2 | 31.9 |  |
|  | Missing | 3.8 | 4.6 | 3.8 | 3.6 | 4.0 |  |
| Nottingham histological grade | Grade 1 | 20.4 | 19.2 | 18.3 | 18.7 | 19.2 | 0.481 |
|  | Grade 2 | 43.8 | 48.7 | 46.9 | 44.9 | 46.1 |  |
|  | Grade 3 | 31.5 | 29.9 | 32.4 | 33.8 | 31.9 |  |
|  | Missing | 4.4 | 2.2 | 2.4 | 2.6 | 2.9 |  |
| Estrogen receptor status | Negative | 13.3 | 11.9 | 13.9 | 17.1 | 14.1 | 0.235 |
|  | Positive | 86.3 | 88.1 | 85.7 | 82.5 | 85.7 |  |
| Progesterone receptor status | Negative | 25.3 | 25.1 | 31.0 | 30.2 | 27.9 | 0.093 |
|  | Positive | 74.3 | 74.9 | 68.8 | 69.2 | 71.8 |  |
| HER2 status | Negative | 85.5 | 88.7 | 84.7 | 85.9 | 86.2 | 0.318 |
|  | Positive | 12.9 | 10.3 | 13.1 | 13.3 | 12.4 |  |
|  | Missing | 1.6 | 1.0 | 2.2 | 0.8 | 1.4 |  |
| Ki67 status | Low | 11.7 | 12.1 | 11.7 | 9.9 | 11.4 | 0.756 |
|  | High | 12.9 | 13.7 | 12.9 | 11.3 | 12.7 |  |
|  | Missing | 75.4 | 74.1 | 75.3 | 78.9 | 75.9 |  |

All data is presented as column percentage. Missing values not presented if <1%.

**Table S3.** Total T4 levels in relation to percentage distribution of tumour characteristics, age, and menopausal status.

| Factor | | Total T4 | | | | |  |
| --- | --- | --- | --- | --- | --- | --- | --- |
|  | | ≤93.19 pmol/L | 93.20 - 104.45 pmol/L | 104.46 - 116.40 pmol/L | >116.41  pmol/L | All | p-value |
|  | | n=500 | n=500 | n=504 | n=496 | n=2000 |  |
| Age at diagnosis | <50 | 9.0 | 8.4 | 9.1 | 9.3 | 9.0 | 0.019 |
|  | 50-60 | 33.4 | 32.4 | 28.8 | 27.4 | 30.5 |  |
|  | 60-70 | 38.2 | 33.2 | 36.7 | 32.1 | 35.1 |  |
|  | >70 | 19.4 | 26.0 | 25.4 | 31.0 | 25.5 |  |
| Menopausal status | Premenopausal | 18.2 | 17.6 | 17.1 | 20.4 | 18.3 | 0.412 |
|  | Postmenopausal | 74.6 | 77.6 | 76.8 | 76.0 | 76.3 |  |
|  | Uncertain | 5.4 | 3.8 | 5.0 | 2.6 | 4.2 |  |
|  | Missing | 1.8 | 1.0 | 1.2 | 1.0 | 1.3 |  |
| Diagnosis by screening mammography | No | 43.6 | 45.2 | 45.2 | 51.6 | 46.4 | 0.128 |
|  | Yes | 55.4 | 54.0 | 53.4 | 46.8 | 52.4 |  |
|  | Missing | 1.0 | 0.8 | 1.4 | 1.6 | 1.2 |  |
| Tumour size | ≤20mm | 70.6 | 70.2 | 67.5 | 63.9 | 68.1 | 0.186 |
|  | >20mm | 28.6 | 28.8 | 31.5 | 35.7 | 31.2 |  |
| Tumour type (histological) | Other + mixed | 7.2 | 5.0 | 8.5 | 6.7 | 6.9 | 0.317 |
|  | Ductal | 78.8 | 81.8 | 78.0 | 81.5 | 80.0 |  |
|  | Lobular | 14.0 | 13.0 | 13.5 | 11.5 | 13.0 |  |
| Lymph node status | Node negative | 62.4 | 66.6 | 66.1 | 61.7 | 64.2 | 0.268 |
|  | Node positive | 34.4 | 28.8 | 29.6 | 34.7 | 31.9 |  |
|  | Missing | 3.2 | 4.6 | 4.4 | 3.6 | 4.0 |  |
| Nottingham histological grade | Grade 1 | 17.4 | 21.2 | 20.2 | 17.7 | 19.2 | 0.313 |
|  | Grade 2 | 50.0 | 45.6 | 45.0 | 433.5 | 46.1 |  |
|  | Grade 3 | 29.8 | 31.0 | 32.1 | 34.7 | 31.9 |  |
|  | Missing | 2.8 | 2.2 | 2.6 | 4.0 | 2.9 |  |
| Estrogen receptor status | Negative | 12.0 | 13.0 | 14.9 | 16.3 | 14.1 | 0.234 |
|  | Positive | 87.6 | 86.8 | 85.1 | 83.1 | 85.7 |  |
| Progesterone receptor status | Negative | 24.8 | 27.4 | 29.6 | 29.8 | 27.9 | 0.281 |
|  | Positive | 74.8 | 72.4 | 70.4 | 69.6 | 71.8 |  |
| HER2 status | Negative | 86.0 | 85.8 | 87.7 | 85.3 | 86.2 | 0.843 |
|  | Positive | 12.6 | 12.6 | 11.5 | 12.9 | 12.4 |  |
|  | Missing | 1.4 | 1.6 | 0.8 | 1.8 | 1.4 |  |
| Ki67 status | Low | 10.0 | 10.6 | 14.1 | 10.7 | 11.4 | 0.288 |
|  | High | 11.0 | 13.4 | 13.1 | 13.3 | 12.7 |  |
|  | Missing | 79.0 | 76.0 | 72.8 | 76.0 | 75.9 |  |

All data is presented as column percentage. Missing values not presented if <1%.

**Table S4.** Free T3/T4 ratio levels in relation to percentage distribution of tumour characteristics, age, and menopausal status.

| Factor | | Free T3/T4 | | | | |  |
| --- | --- | --- | --- | --- | --- | --- | --- |
|  | | Ratio  ≤0.25 | Ratio  0.26-0.28 | Ratio 0.29-0.31 | Ratio  ≥0.32 | All | p-value |
|  | | n=500 | n=500 | n=500 | n=500 | n=2000 |  |
| Age at diagnosis | <50 | 4.4 | 8.8 | 12.6 | 10.0 | 9.0 | 0.000 |
|  | 50-60 | 21.0 | 28.8 | 33.2 | 39.0 | 30.5 |  |
|  | 60-70 | 36.8 | 34.0 | 35.2 | 34.2 | 35.1 |  |
|  | >70 | 37.8 | 28.4 | 18.8 | 16.8 | 25.5 |  |
| Menopausal status | Premenopausal | 12.6 | 17.2 | 22.4 | 21.0 | 18.3 | <0.001 |
|  | Postmenopausal | 83.8 | 77.6 | 71.0 | 72.6 | 76.3 |  |
|  | Uncertain | 2.6 | 4.0 | 5.0 | 5.2 | 4.2 |  |
|  | Missing | 1.0 | 1.2 | 1.6 | 1.2 | 1.3 |  |
| Diagnosis by screening mammography | No | 52.8 | 48.8 | 44.6 | 39.8 | 46.4 | <0.001 |
|  | Yes | 45.4 | 50.8 | 53.6 | 59.8 | 52.4 |  |
|  | Missing | 1.8 | 0.8 | 1.8 | 0.4 | 1.2 |  |
| Tumour size | ≤20mm | 66.8 | 66.8 | 69.4 | 69.2 | 68.1 | 0.906 |
|  | >20mm | 32.6 | 32.4 | 29.6 | 30.0 | 31.2 |  |
| Tumour type (histological) | Other + mixed | 8.6 | 6.4 | 5.2 | 7.2 | 6.9 | 0.355 |
|  | Ductal | 78.0 | 78.8 | 81.0 | 82.2 | 80.0 |  |
|  | Lobular | 13.4 | 14.6 | 13.6 | 10.4 | 13.0 |  |
| Lymph node status | Node negative | 63.2 | 64.0 | 64.2 | 65.4 | 64.2 | 0.997 |
|  | Node positive | 32.8 | 32.0 | 31.8 | 30.8 | 31.9 |  |
|  | Missing | 4.0 | 4.0 | 4.0 | 3.8 | 4.0 |  |
| Nottingham histological grade | Grade 1 | 20.2 | 17.6 | 18.6 | 20.2 | 19.2 | 0.823 |
|  | Grade 2 | 47.0 | 47.0 | 45.2 | 45.0 | 46.1 |  |
|  | Grade 3 | 30.4 | 32.6 | 32.2 | 32.4 | 31.9 |  |
|  | Missing | 2.4 | 2.8 | 4.0 | 2.4 | 2.9 |  |
| Estrogen receptor status | Negative | 15.6 | 13.2 | 13.8 | 13.6 | 14.1 | 0.757 |
|  | Positive | 84.2 | 86.6 | 85.6 | 86.2 | 85.7 |  |
| Progesterone receptor status | Negative | 29.8 | 29.6 | 26.2 | 26.0 | 27.9 | 0.525 |
|  | Positive | 70.0 | 70.2 | 73.2 | 73.8 | 71.8 |  |
| HER2 status | Negative | 87.4 | 83.0 | 86.6 | 87.8 | 86.2 | 0.344 |
|  | Positive | 11.6 | 15.4 | 11.8 | 10.8 | 12.4 |  |
|  | Missing | 1.0 | 1.6 | 1.6 | 1.4 | 1.4 |  |
| Ki67 status | Low | 12.6 | 9.2 | 11.2 | 12.4 | 11.4 | 0.599 |
|  | High | 11.6 | 12.8 | 13.8 | 12.6 | 12.7 |  |
|  | Missing | 75.8 | 78.0 | 75.0 | 75.0 | 75.9 |  |

All data is presented as column percentage. Missing values not presented if <1%.

**Table S5.** Free T3 levels in relation percentage distribution of adjuvant therapy and surgical procedure.

| Treatment |  |  | Free T3 | | |  |
| --- | --- | --- | --- | --- | --- | --- |
|  |  | ≤4.20 pmol/L | 4.21 - 4.56 pmol/L | 4.57 - 4.95 pmol/L | >4.96 pmol/L | Total |
|  |  | n=510 | n=501 | n=490 | n=499 | n=2000 |
| Endocrine | No | 25.6 | 25.2 | 26.9 | 26.4 | 26.0 |
|  | Yes | 74.4 | 74.8 | 73.1 | 73.6 | 74.0 |
| Radiotherapy | No | 40.7 | 36.2 | 29.6 | 29.2 | 34.0 |
|  | Yes | 59.3 | 63.8 | 70.4 | 70.8 | 66.0 |
| Chemotherapy | No | 73.2 | 66.0 | 67.6 | 57.3 | 66.1 |
|  | Yes | 26.8 | 34.0 | 32.4 | 42.7 | 33.9 |
| Immunotherapy | No | 90.6 | 88.8 | 89.7 | 89.1 | 89.6 |
|  | Yes | 9.4 | 11.2 | 10.3 | 10.9 | 10.4 |
| Breast surgery | No surgery | 0.0 | 0.0 | 0.0 | 0.0 | 0.0 |
|  | Mastectomy | 46.7 | 41.7 | 37.6 | 38.8 | 41.2 |
|  | Partial mastectomy | 53.3 | 58.3 | 62.4 | 61.2 | 58.8 |
| Axillary surgery | No surgery | 1.0 | 0.4 | 0.2 | 0.2 | 0.5 |
|  | SN/ sampling | 66.1 | 65.8 | 64.3 | 63.2 | 64.8 |
|  | Clearance | 32.9 | 33.8 | 35.5 | 36.6 | 34.7 |

All data is presented as column percentage. Missing values not presented if <1%.

**Table S6.** Total T3 levels in relation percentage distribution of adjuvant therapy and surgical procedure.

| Treatment |  |  | Total T3 | | |  |
| --- | --- | --- | --- | --- | --- | --- |
|  |  | ≤1.36  pmol/L | 1.36 - 1.53 pmol/L | 1.53 - 1.72 pmol/L | >1.72  pmol/L | All |
|  |  | n=513 | n=511 | n=494 | n=482 | n=2000 |
| Endocrine | No | 24.1 | 24.4 | 28.7 | 27.0 | 26.0 |
|  | Yes | 75.9 | 75.6 | 71.3 | 73.0 | 74.0 |
| Radiotherapy | No | 38.2 | 36.9 | 32.6 | 27.9 | 34.0 |
|  | Yes | 61.8 | 63.1 | 67.4 | 72.1 | 66.0 |
| Chemotherapy | No | 72.0 | 66.8 | 65.0 | 60.1 | 66.1 |
|  | Yes | 28.0 | 33.2 | 35.0 | 39.9 | 33.9 |
| Immunotherapy | No | 90.0 | 90.4 | 89.0 | 88.8 | 89.6 |
|  | Yes | 10.0 | 9.6 | 11.0 | 11.2 | 10.4 |
| Breast surgery | No surgery | 0.0 | 0.0 | 0.0 | 0.0 | 0.0 |
|  | Mastectomy | 43.9 | 42.1 | 42.0 | 36.7 | 41.2 |
|  | Partial mastectomy | 56.1 | 57.9 | 58.0 | 63.3 | 58.8 |
| Axillary surgery | No surgery | 0.4 | 1.0 | 0.4 | 0.0 | 0.5 |
|  | SN/ sampling | 65.8 | 62.2 | 65.3 | 66.1 | 64.8 |
|  | Clearance | 33.8 | 36.8 | 34.3 | 33.9 | 34.7 |

All data is presented as column percentage. Missing values not presented if <1%.

**Table S7.** Free T4 levels in relation percentage distribution of adjuvant therapy and surgical procedure.

| Treatment |  |  | Free T4 | | |  |
| --- | --- | --- | --- | --- | --- | --- |
|  |  | ≤14.97 pmol/L | 14.98 - 16.49 pmol/L | 16.51 - 18.33 pmol/L | >18.34 pmol/L | Total |
|  |  | n=505 | n=495 | n=503 | n=497 | n=2000 |
| Endocrine | No | 25.3 | 23.6 | 26.4 | 28.7 | 26.0 |
|  | Yes | 74.7 | 76.4 | 73.6 | 71.3 | 74.0 |
| Radiotherapy | No | 30.1 | 32.5 | 37.4 | 36.0 | 34.0 |
|  | Yes | 69.9 | 67.5 | 62.6 | 64.0 | 66.0 |
| Chemotherapy | No | 63.8 | 66.1 | 66.4 | 68.1 | 66.1 |
|  | Yes | 36.2 | 33.9 | 33.6 | 31.9 | 33.9 |
| Immunotherapy | No | 88.5 | 91.3 | 88.1 | 90.4 | 89.6 |
|  | Yes | 11.5 | 8.7 | 11.9 | 9.6 | 10.4 |
| Breast surgery | No surgery | 0.0 | 0.0 | 0.0 | 0.0 | 0.0 |
|  | Mastectomy | 37.0 | 41.0 | 41.0 | 46.0 | 41.2 |
|  | Partial mastectomy | 63.0 | 59.0 | 59.0 | 54.0 | 58.8 |
| Axillary surgery | No surgery | 0.4 | 0.8 | 0.4 | 0.2 | 0.5 |
|  | SN/ sampling | 64.5 | 65.7 | 68.8 | 60.4 | 64.8 |
|  | Clearance | 35.1 | 33.5 | 30.8 | 39.4 | 34.7 |

All data is presented as column percentage. Missing values not presented if <1%.

**Table S8.** Total T4 levels in relation percentage distribution of adjuvant therapy and surgical procedure.

| Treatment |  |  | Total T4 | | |  |
| --- | --- | --- | --- | --- | --- | --- |
|  |  | ≤93.19 pmol/L | 93.20 - 104.45 pmol/L | 104.46 - 116.40 pmol/L | >116.41 pmol/L | Total |
|  |  | n=500 | n=500 | n=504 | n=496 | n=2000 |
| Endocrine | No | 23.4 | 26.9 | 26.3 | 27.3 | 26.0 |
|  | Yes | 76.6 | 73.1 | 73.7 | 72.7 | 74.0 |
| Radiotherapy | No | 31.5 | 33.3 | 37.3 | 33.8 | 34.0 |
|  | Yes | 68.5 | 66.7 | 62.7 | 66.2 | 66.0 |
| Chemotherapy | No | 63.1 | 66.7 | 69.1 | 65.4 | 66.1 |
|  | Yes | 36.9 | 33.3 | 30.9 | 34.6 | 33.9 |
| Immunotherapy | No | 88.8 | 89.6 | 90.4 | 89.5 | 89.6 |
|  | Yes | 11.2 | 10.4 | 9.6 | 10.5 | 10.4 |
| Breast surgery | No surgery | 0.0 | 0.0 | 0.0 | 0.0 | 0.0 |
|  | Mastectomy | 39.8 | 39.2 | 40.7 | 45.3 | 41.2 |
|  | Partial mastectomy | 60.2 | 60.8 | 59.3 | 54.7 | 58.8 |
| Axillary surgery | No surgery | 0.4 | 0.4 | 1.0 | 0.0 | 0.5 |
|  | SN/ sampling | 63.6 | 66.9 | 66.9 | 61.9 | 64.8 |
|  | Clearance | 36.0 | 32.7 | 32.1 | 38.1 | 34.7 |

All data is presented as column percentage. Missing values not presented if <1%.

**Table S9.** Free T3/T4 levels in relation percentage distribution of adjuvant therapy and surgical procedure.

| Treatment |  |  | Free T3/T4 | | |  |
| --- | --- | --- | --- | --- | --- | --- |
|  |  | Ratio  ≤0.25 | Ratio  0.26 – 0.28 | Ratio  0.29 – 0.31 | Ratio ≥0.32 | Total |
|  |  | n=500 | n=500 | n=500 | n=500 | n=2000 |
| Endocrine | No | 26.8 | 23.8 | 24.2 | 27.2 | 25.9 |
|  | Yes | 70.6 | 76.2 | 75.4 | 72.6 | 73.7 |
| Radiotherapy | No | 43.2 | 33.8 | 29.8 | 28.6 | 33.9 |
|  | Yes | 56.0 | 66.0 | 69.8 | 71.2 | 65.8 |
| Chemotherapy | No | 74.6 | 64.8 | 61.2 | 62.6 | 65.8 |
|  | Yes | 24.6 | 35.0 | 38.4 | 37.2 | 33.8 |
| Immunotherapy | No | 91.0 | 87.4 | 89.6 | 88.8 | 89.2 |
|  | Yes | 8.2 | 12.4 | 10.0 | 11.0 | 10.4 |
| Breast surgery | No surgery | 0.0 | 0.0 | 0.0 | 0.0 | 0.0 |
|  | Mastectomy | 50.0 | 41.0 | 38.6 | 35.2 | 41.2 |
|  | Partial mastectomy | 50.0 | 59.0 | 61.2 | 64.8 | 41.2 |
| Axillary surgery | No surgery | 0.8 | 0.6 | 0.2 | 0.2 | 0.5 |
|  | SN/ sampling | 63.0 | 66.0 | 65.0 | 65.0 | 64.8 |
|  | Clearance | 36.2 | 33.2 | 34.4 | 34.8 | 34.7 |

All data is presented as column percentage. Missing values not presented if <1%.

**Table S10.** Vital status at end of follow up in relation to percentage distribution of tumour characteristics, age, and menopausal status.

| Factor |  | Vital status at end of follow-up | | |  |
| --- | --- | --- | --- | --- | --- |
|  |  | Alive | Deceased | All | p-value |
|  |  | n=1690 | n=310 | 2000 |  |
| Age at diagnosis | <50 | 9.8 | 4.5 | 9.0 | <0.001 |
|  | 50-60 | 33.7 | 12.9 | 30.5 |  |
|  | 60-70 | 36.0 | 30.0 | 35.1 |  |
|  | >70 | 20.5 | 52.6 | 25.5 |  |
| Menopausal status | Premenopausal | 20.3 | 7.4 | 18.3 | <0.001 |
|  | Postmenopausal | 73.8 | 89.7 | 76.3 |  |
|  | Uncertain | 4.7 | 1.3 | 4.2 |  |
|  | Missing | 1.2 | 1.6 | 1.3 |  |
| Diagnosis by screening mammography | No | 42.8 | 66.1 | 46.4 | <0.001 |
|  | Yes | 56.9 | 33.5 | 52.4 |  |
|  | Missing | 1.4 | 0.3 | 1.2 |  |
| Tumour size | ≤20mm | 72.2 | 45.5 | 68.1 | <0.001 |
|  | >20mm | 27.0 | 53.5 | 31.2 |  |
| Tumour type (histological) | Other + mixed | 6.3 | 9.7 | 6.9 | 0.164 |
|  | Ductal | 80.4 | 77.7 | 80.0 |  |
|  | Lobular | 13.1 | 12.6 | 13.0 |  |
| Lymph node status | Node negative | 65.3 | 58.4 | 64.2 | 0.057 |
|  | Node positive | 31.0 | 36.5 | 31.9 |  |
|  | Missing | 3.7 | 5.2 | 4.0 |  |
| Nottingham histological grade | Grade 1 | 20.8 | 10.3 | 19.2 | <0.001 |
|  | Grade 2 | 46.9 | 41.3 | 46.1 |  |
|  | Grade 3 | 29.7 | 43.9 | 31.9 |  |
|  | Missing | 2.6 | 4.5 | 2.9 |  |
| Estrogen receptor status | Negative | 11.9 | 25.8 | 14.1 | <0.001 |
|  | Positive | 87.8 | 73.9 | 85.7 |  |
| Progesterone receptor status | Negative | 25.1 | 43.2 | 27.9 | <0.001 |
|  | Positive | 74.6 | 56.8 | 71.8 |  |
| HER2 status | Negative | 86.7 | 83.5 | 86.2 | 0.037 |
|  | Positive | 12.2 | 13.5 | 12.4 |  |
|  | Missing | 1.1 | 2.9 | 1.4 |  |
| Ki67 status | Low | 12.4 | 5.8 | 11.4 | 0.003 |
|  | High | 12.7 | 12.9 | 12.7 |  |
|  | Missing | 75.0 | 81.3 | 75.9 |  |

All data is presented as column percentage. Missing values not presented if <1%.

**Table S11.** Non-participants as compared to participants in relation to

percentage distribution of tumour characteristics, age, and menopausal status.

| Factor |  | Participants | | |
| --- | --- | --- | --- | --- |
|  |  | No | Yes | Total |
|  |  | n= 903 | n=2000 | n=2903 |
| Age at diagnosis | <50 | 9.0 | 9.0 | 9.0 |
|  | 50-60 | 32.0 | 30.5 | 31.0 |
|  | 60-70 | 32.2 | 35.1 | 34.2 |
|  | >70 | 25.2 | 25.5 | 25.4 |
| Menopausal status | Premenopausal | 19.7 | 18.3 | 18.7 |
|  | Postmenopausal | 72.6 | 76.3 | 75.1 |
|  | Uncertain | 5.4 | 4.2 | 4.6 |
|  | Missing | 2.2 | 1.3 | 1.6 |
| Diagnosis by screening mammography | No | 38.2 | 46.4 | 43.9 |
|  | Yes | 60.2 | 52.4 | 54.8 |
|  | Missing | 1.6 | 1.2 | 1.3 |
| Tumour size | ≤20mm | 72.1 | 68.1 | 69.3 |
|  | >20mm | 26.1 | 31.2 | 29.6 |
|  | Missing | 1.8 | 0.8 | 1.1 |
| Tumour type (histological) | Other + mixed | 7.6 | 6.9 | 7.1 |
|  | Ductal | 78.1 | 80.0 | 79.4 |
|  | Lobular | 12.4 | 13.0 | 12.8 |
|  | Missing | 1.9 | 0.2 | 0.7 |
| Lymph node status | Node negative | 65.7 | 64.2 | 64.7 |
|  | Node positive | 29.3 | 31.9 | 31.1 |
|  | Missing | 5.0 | 4.0 | 4.3 |
| Nottingham histological grade | Grade 1 | 19.2 | 19.2 | 19.2 |
|  | Grade 2 | 47.0 | 46.1 | 46.3 |
|  | Grade 3 | 30.3 | 31.9 | 31.4 |
|  | Missing | 3.5 | 2.9 | 3.1 |
| Estrogen receptor status | Negative | 12.2 | 14.1 | 13.5 |
|  | Positive | 85.5 | 85.7 | 85.6 |
|  | Missing | 2.3 | 0.3 | 0.9 |
| Progesterone receptor status | Negative | 22.8 | 27.9 | 26.3 |
|  | Positive | 74.8 | 71.8 | 72.7 |
|  | Missing | 2.4 | 0.3 | 1.0 |
| HER2 status | Negative | 85.2 | 86.2 | 85.9 |
|  | Positive | 11.8 | 12.4 | 12.2 |
|  | Missing | 3.0 | 1.4 | 1.9 |
| Ki67 status | Low | 3.0 | 11.4 | 8.7 |
|  | High | 2.8 | 12.7 | 9.6 |
|  | Missing | 94.2 | 75.9 | 81.6 |

All data is presented as column percentage. Missing values not presented if <1%.

**Table S12.** Non-participants as compared to participants in relation to percentage distribution of adjuvant therapy and surgical procedure.

| Treatment |  |  | Participants |  |
| --- | --- | --- | --- | --- |
|  |  | No | Yes | All |
|  |  | n= 903 | n= 2000 | n= 2903 |
| Endocrine | No | 26.5 | 25.9 | 26.1 |
|  | Yes | 71.8 | 73.7 | 73.1 |
|  | Missing | 1.8 | 0.4 | 0.8 |
| Radiotherapy | No | 41.5 | 33.9 | 36.2 |
|  | Yes | 56.7 | 65.8 | 62.9 |
|  | Missing | 1.8 | 0.4 | 0.8 |
| Chemotherapy | No | 67.6 | 65.8 | 66.3 |
|  | Yes | 30.7 | 33.8 | 32.8 |
|  | Missing | 1.8 | 0.4 | 0.8 |
| Immunotherapy | No | 89.1 | 89.2 | 89.2 |
|  | Yes | 9.1 | 10.4 | 10.0 |
|  | Missing | 1.8 | 0.4 | 0.8 |
| Breast surgery | No surgery | 0.1 | 0.0 | 0.0 |
|  | Mastectomy | 49.4 | 41.2 | 43.7 |
|  | Partial mastectomy | 48.9 | 58.8 | 55.7 |
| Axillary surgery | No surgery | 1.4 | 0.5 | 0.8 |
|  | SN/ sampling | 61.4 | 64.8 | 63.7 |
|  | Clearance | 35.0 | 34.7 | 34.8 |
|  | Missing | 2.2 | 0.2 | 0.8 |

All data is presented as column percentage. Missing values not presented if <1%.

**Table S13.** Thyroid hormones and all-cause mortality in postmenopausal patients.

| Hormone | Quartile | Patients (n) | Dead  (n) | Mortality  /100 person years | HR^1^ | HR^2^ | HR^3^ |
| --- | --- | --- | --- | --- | --- | --- | --- |
| Free T3 | 1 | 421 | 103 | 3.83 | 1 | 1 | 1 |
|  | 2 | 380 | 64 | 2.53 | 0.66  (0.48 - 0.90) | 0.72  (0.53 - 0.98) | 0.75  (0.55 - 1.03) |
|  | 3 | 368 | 53 | 2.12 | 0.55  (0.39 - 0.76) | 0.70  (0.50 - 0.97) | 0.69  (0.49 - 0.97) |
|  | 4 | 356 | 58 | 2.47 | 0.64  (0.47 - 0.87) | 0.84  (0.60 - 1.16) | 0.76  (0.55 - 1.06) |
|  |  |  |  |  |  |  |  |
| Free T4 | 1 | 374 | 64 | 2.57 | 1 | 1 | 1 |
|  | 2 | 372 | 61 | 2.45 | 0.96  (0.67 - 1.36) | 0.95  (0.67 - 1.35) | 0.96  (0.67 - 1.36) |
|  | 3 | 385 | 55 | 2.12 | 0.83  (0.58 - 1.19) | 0.77  (0.54 - 1.11) | 0.73  (0.51 - 1.05) |
|  | 4 | 394 | 98 | 3.92 | 1.54  (1.12 - 2.11) | 1.38  (1.00 - 1.89) | 1.31  (0.95 - 1.80) |
|  |  |  |  |  |  |  |  |
| Total T3 | 1 | 404 | 86 | 3.28 | 1 | 1 | 1 |
|  | 2 | 404 | 70 | 2.59 | 0.78  (0.57 - 1.08) | 0.86  (0.63 - 1.18) | 0.86  (0.63 - 1.19) |
|  | 3 | 372 | 65 | 2.67 | 0.81  (0.59 - 1.12) | 0.99  (0.72 - 1.38) | 0.97  (0.70 - 1.34) |
|  | 4 | 345 | 57 | 2.48 | 0.76  (0.54 - 1.06) | 0.97  (0.69 - 1.36) | 0.86  (0.61 - 1.21) |
|  |  |  |  |  |  |  |  |
| Total T4 | 1 | 373 | 58 | 2.33 | 1 | 1 | 1 |
|  | 2 | 388 | 59 | 2.25 | 0.97  (0.67 - 1.39) | 0.88  (0.62 - 1.27) | 0.93  (0.65 - 1.34) |
|  | 3 | 387 | 68 | 2.66 | 1.15  (0.81 - 1.63) | 1.04  (0.73 - 1.48) | 0.98  (0.69 - 1.40) |
|  | 4 | 377 | 93 | 3.88 | 1.68  (1.20 - 2.33) | 1.41  (1.01 - 1.96) | 1.28  (0.91 - 1.78) |
|  |  |  |  |  |  |  |  |
| Free T3/Free T4 | 1 | 419 | 109 | 4.15 | 1 | 1 | 1 |
|  | 2 | 388 | 64 | 2.45 | 0.58  (0.43 - 0.79) | 0.63  (0.47 - 0.86) | 0.64  (0.47 - 0.88) |
|  | 3 | 355 | 51 | 2.13 | 0.51  (0.36 - 0.71) | 0.63  (0.45 - 0.88) | 0.64  (0.45 - 0.90) |
|  | 4 | 363 | 54 | 2.23 | 0.53  (0.38 - 0.74) | 0.69  (0.49 - 0.96) | 0.71  (0.51 - 0.99) |
|  |  |  |  |  |  |  |  |

^1^ crude analysis. ^2^ adjusted for age. ^3^ adjusted for age, tumor size, diagnosis by screening mammography, estrogen receptor status, progesterone receptor status, HER2-receptor status, Nottingham histological grade, lymph node status and histological tumor type.

| Hormone | Quartile | Patients  (n) | Dead  (n) | Mortality  /100 person years | HR^1^ | HR^2^ | HR^3^ |
| --- | --- | --- | --- | --- | --- | --- | --- |
| Free T3 | 1 | 510 | 47 | 1.46 | 1 | 1 | 1 |
|  |  |  |  |  |  |  |  |
|  | 2 | 501 | 39 | 1.17 | 0.81  (0.53-1.24) | 0.83  (0.54-1.27) | 0.83  (0.54-1.28) |
|  |  |  |  |  |  |  |  |
|  | 3 | 490 | 36 | 1.16 | 0.76  (0.49-1.17) | 0.83  (0.53-1.29) | 0.82  (0.53-1.28) |
|  |  |  |  |  |  |  |  |
|  | 4 | 498 | 45 | 1.37 | 0.96  (0.64-1.44) | 1.05  (0.69-1.59) | 0.94  (0.62-1.43) |
|  |  |  |  |  |  |  |  |
| Total T3 | 1 | 513 | 42 | 1.27 | 1 | 1 | 1 |
|  |  |  |  |  |  |  |  |
|  | 2 | 511 | 38 | 1.12 | 0.86  (0.57-1.37) | 0.92  (0.59-1.43) | 0.90  (0.58-1.40) |
|  |  |  |  |  |  |  |  |
|  | 3 | 493 | 37 | 1.15 | 0.91  (0.58-1.41) | 1.00  (0.64-1.55) | 0.91  (0.58-1.43) |
|  |  |  |  |  |  |  |  |
|  | 4 | 482 | 50 | 1.59 | 1.25  (0.83-1.89) | 1.38  (0.91-2.09) | 1.20  (0.79-1.83) |
| Free T4 | 1 | 505 | 44 | 1.32 | 1 | 1 | 1 |
|  |  |  |  |  |  |  |  |
|  | 2 | 495 | 37 | 1.14 | 0.85  (0.55-1.32) | 0.85  (0.55-1.32) | 0.91  (0.58-1.41) |
|  |  |  |  |  |  |  |  |
|  | 3 | 503 | 40 | 1.19 | 0.90  (0.58-1.37) | 0.87  (0.57-1.34) | 0.86  (0.56-1.32) |
|  |  |  |  |  |  |  |  |
|  | 4 | 496 | 46 | 1.46 | 1.09  (0.72-1.65) | 1.05  (0.69-1.58) | 0.99  (0.65-1.51) |
| Total T4 | 1 | 500 | 38 | 1.15 | 1 | 1 | 1 |
|  |  |  |  |  |  |  |  |
|  | 2 | 500 | 43 | 1.30 | 1.13  (0.73-1.75) | 1.10  (0.71-1.70) | 1.10  (0.71-1.71) |
|  |  |  |  |  |  |  |  |
|  | 3 | 504 | 36 | 1.09 | 0.94  (0.60-1.48) | 0.90  (0.57-1.42) | 0.85  (0.54-1.35) |
|  |  |  |  |  |  |  |  |
|  | 4 | 495 | 50 | 1.59 | 1.37  (0.90-2.10) | 1.27  (0.83-1.95) | 1.15  (0.75-1.77) |
| Free T3/free T4 | 1 | 500 | 50 | 1.61 | 1 | 1 | 1 |
|  |  |  |  |  |  |  |  |
|  | 2 | 500 | 33 | 0.99 | 0.62  (0.40-0.96) | 0.63  (0.41-0.98) | 0.63  (0.40-0.98) |
|  |  |  |  |  |  |  |  |
|  | 3 | 500 | 37 | 1.11 | 0.70  (0.46-1.10) | 0.75  (0.48-1.15) | 0.74  (0.48-1.15) |
|  |  |  |  |  |  |  |  |
|  | 4 | 500 | 47 | 1.43 | 0.90  (0.60-1.34) | 0.99  (0.66-1.49) | 0.99  (0.66-1.50) |

**Table S14.** Thyroid hormones and recurrent disease.

^1^ crude analysis. ^2^ adjusted for age. ^3^ adjusted for age, menopause status, tumor size, diagnosis by screening mammography, estrogen receptor status, progesterone receptor status, HER2-receptor status, Nottingham histological grade, lymph node status and histological tumor type.
