# Supplementary material for: Serum Levels of Thyroid Hormones in Relation to Breast Cancer Prognosis: A Multicentre Cohort Study
Source: Cancer Med. 2026 Sep 9;15(9):e72264. doi: 10.1002/cam4.72264 (PMC13559021; doi:10.1002/cam4.72264)
Supplement: Supplementary file 3 [file CAM4-15-e72264-s003.docx]

Figure S1. Selected Schoenfeld residual plots for checking the proportional hazards assumption. The plots indicate that no violations of the proportional hazards assumption were observed.
