# Supplementary material for: Serum Levels of Thyroid Hormones in Relation to Breast Cancer Prognosis: A Multicentre Cohort Study
Source: Cancer Med. 2026 Sep 9;15(9):e72264. doi: 10.1002/cam4.72264 (PMC13559021; doi:10.1002/cam4.72264)
Supplement: Supplementary file 4 [file CAM4-15-e72264-s004.docx]

Figure S2. Total T3 (a) and Total T4 (b) in relation to survival following a breast cancer diagnosis, using all-cause mortality as endpoint.

**a**

p=0.348

**a**

p<0.01

**b**
